# Unintended consequences: a qualitative study exploring the impact of collecting implementation process data with phone interviews on implementation activities

**DOI:** 10.1186/s43058-020-00093-7

**Published:** 2020-11-04

**Authors:** Inga Gruß, Arwen Bunce, James Davis, Rachel Gold

**Affiliations:** 1 Kaiser Permanente Center for Health Research, 3800 N. Interstate, Portland, OR 97227 USA; 2 OCHIN, Inc., 1881 SW Naito Pkwy, Portland, OR 97201 USA

**Keywords:** Capturing implementation processes, Implementation studies, Data collection methods, Study design, Researcher effects, Measurement reactivity, Qualitative research

## Abstract

**Background:**

Qualitative data are crucial for capturing implementation processes, and thus necessary for understanding implementation trial outcomes. Typical methods for capturing such data include observations, focus groups, and interviews. Yet little consideration has been given to how such methods create interactions between researchers and study participants, which may affect participants’ engagement, and thus implementation activities and study outcomes. In the context of a clinical trial, we assessed whether and how ongoing telephone check-ins to collect data about implementation activities impacted the quality of collected data, and participants’ engagement in study activities.

**Methods:**

Researchers conducted regular phone check-ins with clinic staff serving as implementers in an implementation study. Approximately 1 year into this trial, 19 of these study implementers were queried about the impact of these calls on study engagement and implementation activities. The two researchers who collected implementation process data through phone check-ins with the study implementers were also interviewed about their perceptions of the impact of the check-ins.

**Results:**

Study implementers’ assessment of the check-ins’ impact fell into three categories: (1) the check-ins had no effect on implementation activities, (2) the check-ins served as a reminder about study participation (without relating a clear impact on implementation activities), and (3) the check-ins caused changes in implementation activities. The researchers similarly perceived that the phone check-ins served as reminders and encouraged some implementers’ engagement in implementation activities; their ongoing nature also created personal connections with study implementers that may have impacted implementation activities. Among some study implementers, anticipation of the check-in calls also improved their ability to recount implementation activities and positively affected quality of the data collected.

**Conclusion:**

These results illustrate the potential impact of qualitative data collection on implementation activities during implementation science trials. Mitigating such effects may prove challenging, but acknowledging these consequences—or even embracing them, perhaps by designing data collection methods as implementation strategies—could enhance scientific rigor. This work is presented to stimulate debate about the complexities involved in capturing data on implementation processes using common qualitative data collection methods.

**Trial registration:**

ClinicalTrials.gov, NCT02325531. Registered 15 December 2014.

Contributions to the literature

- Qualitative data are widely collected in implementation science trials to understand implementation processes and outcomes, but critical discourse about the potential unintended consequences of such methods is limited.
- We found that regularly scheduled data collection phone check-ins impacted study implementers’ awareness of study participation and implementation activities.
- These findings point to the need for robust dialogue about qualitative data collection methods in implementation science to determine how such data can be captured in a minimally impactful way, and/or if qualitative data collection methods may also be designed to serve as implementation strategies.

## Background

Social scientists have long reported on how study participants’ interactions with researchers can impact participant behaviors [1–4]. In health services research, assessments of researcher–participant interactions have primarily focused on the Hawthorne effect: behavior change among individuals when observed by others. The Hawthorne effect was originally described in the context of factory workers’ performance when observed by supervisors [5, 6]. Since then, it has been applied to describe a variety of changes in patients’ [7] and professionals’ behavior when under observation [8, 9]. However, the conditions under which the Hawthorne effect operates, its specific operating mechanisms, and its effect size are not well-specified [10–14]; as a result, it is poorly defined and applied indiscriminately [13]. Alternative concepts that have emerged to assess measurement reactivity include the question–behavior effect which captures changes that occur after being prompted to reflect on behavior intentions or predictions [15]. Novel methodological approaches, such as incorporating the perspectives of both researchers and participants, may also help elucidate how and when interactions between researchers and participants affect study results [13, 16, 17].

In implementation science studies, researchers and participants often interact as researchers collect data on factors impacting interventions’ adoption [18–22]. Such data provides critical information on organizational contexts, dynamic changes, and stakeholder perspectives [23–25]. Typical qualitative methods for capturing implementation process data include observations, focus groups, interviews, and tracking logs [26–28]. However, in implementation science, little consideration has previously been given to the impact of using such methods on study participants’ engagement with the study team, their clinic-based implementation activities, and study outcomes. To better understand these potential impacts, we interviewed researchers and study participants involved in an implementation science trial called “SpreadNet.” This study of the relative impact of increasingly intensive implementation support on adoption of cardioprotective, guideline-concordant prescribing was an ideal setting in which to assess whether and how ongoing phone check-ins designed to capture qualitative data about implementation activities impacted the quality of the data collected, participants’ engagement in the study, and relevant clinic activities.

## Methods

### Research setting

The parent study, “SpreadNet,” compared the effectiveness of scalable strategies for supporting community health centers’ (CHCs) adoption of a suite of clinical decision support tools for cardiovascular disease (CVD) management. These electronic health record (EHR)-embedded, guideline-based tools, collectively called the “CVD Bundle,” were designed to improve rates of guideline-concordant cardioprotective prescribing [29]. Twenty-nine clinics managed by 12 CHCs in six states participated; all were members of OCHIN, Inc. (formerly the Oregon Community Health Information Network), a non-profit organization based in Portland, OR, that provides a shared Epic© EHR to > 600 US CHCs. Participating CHCs were randomly assigned to one of three study arms; each arm received increasingly intensive support for implementing the CVD Bundle. Clinics in all arms received an implementation toolkit and a webinar training; clinics in arm 2 also received a 2-day, in-person training; and clinics in arm 3 received toolkit, webinars, and the in-person training, as well as remote and face-to-face practice facilitation. The primary outcome of the trial was the rate of guideline-concordant cardioprotective prescribing across arms [30]. The Standards for Reporting Qualitative Research (SRQR) was used to guide the reporting of qualitative findings. The study was approved by the Kaiser Permanente Northwest Institutional Review Board.

Each CHC in the parent study assigned one or more staff members to be the main point of contact with the study team—the “study implementers.” A given CHC was allowed to appoint one study implementer for up to three clinics. These individuals were expected to support and encourage any study-related activities their clinics implemented. They also acted as liaisons between the clinic and the research team: they participated in phone check-ins at which the research team collected implementation process data and, if randomized to arm 3, coordinated the site visits for practice facilitation. At some clinics, the study implementers changed during the study period because of staff turnover. In total, 30 individuals served as study implementers across the 29 study clinics.

### Data collection

One of three qualitative researchers called each study implementer twice monthly for 6 months following the start of the intervention (August 2015 to January 2016) to document implementation experiences and the perspectives of study clinic staff. For the next year (February 2016 to January 2017), phone check-ins occurred monthly; the year after (February 2017 to June 2018), quarterly. In total, 413 data collection phone check-ins were conducted between August 2015 and June 2018. Phone check-ins were loosely based on a guide, but were designed to be flexible to capture a ground-level view of implementation processes including logistics, surprises, challenges, and solutions. The interview guide covered implementation activities planned or in process, time spent on past activities, staff perceptions and awareness of the CVD Bundle, and contextual factors that might impact implementation. All phone check-ins were recorded with permission and professionally transcribed for analysis.

As the study progressed, some of the interactions between the study team and implementers led the team to wonder whether the phone check-ins might be influencing implementation activities. Thus, in June 2016, the team introduced a question to capture relevant data: “Do you think if these calls hadn’t been part of the study process that awareness or clinic activity would have been different—and if so, how?” Implementers were only asked this question if they had been in that role for more than 6 months. The goal was to ask each implementer this question once by the end of study month 32 (December 2016); 19 of the 20 eligible study implementers were queried; one who took on the role later was not interviewed, as they had been in the role for under 6 months.

After the data collection check-in calls with the implementers ended, a qualitative researcher (IG) who had not been involved in data collection for the parent study interviewed the two remaining members of the original qualitative study team (AB and JD) about their perceptions of how the data collection phone check-ins may have impacted study implementer engagement, the data collected, and resulting outcomes. Combined, these interviewees had conducted calls with 25 of the 30 implementers throughout the data collection period. The interviews were recorded and transcribed with permission. Researchers reviewed the transcripts and reviewed the original audio files for clarification if appropriate.

### Data analysis

In the parent study, a coding dictionary was drafted, reviewed, and revised by the qualitative team conducting the data collection phone check-ins; coding was then applied to a sample of transcripts, with results compared to identify disparate coding decisions. Disagreements were resolved through discussion, and the coding dictionary was revised. The final dictionary included a code that captured all data relevant to understanding the impact of interactions between researchers and implementers: “impact of data collection on clinic implementation activities.” Data from all interviews was coded using QSR NVivo software. Coding was guided by the constant comparative method [31, 32].

Analyses for this manuscript built on those in the parent study by drawing on interviews collected with the same participants. While the parent study analyses focused on the main study outcomes [30], those presented here focused on implementers’ answers to the question about the impact of phone check-ins. We conducted a content analysis [33] to assess potential impacts related to implementation activities, which included either (1) study engagement (degree to which implementers were interacting with researchers through phone calls and webinars) or (2) clinic activities conducted to further the uptake of the CVD Bundle. After initial review of data from responses to this question, four sub-codes were created and applied to identify perceived degree of impact of the phone check-ins: (1) no change, (2) heightened awareness with unclear relationship to action, (3) reminders that impacted unspecified implementation-related activities, and (4) specific implementation-related activities occurring as a result of the calls. The three researchers applied these codes independently, then compared their coding decisions. As it was difficult to define unspecific versus specific implementation-related activities, these categories were collapsed into one code that included any descriptions of study-related activities occurring in response to the check-ins. The researchers’ perceptions of the check-in calls’ impact on study engagement, implementation activities, and any other observed effects were assessed by coding for “described effect of calls” in the two relevant transcripts. Data from the study team member interviews were coded by IG.

## Results

### Study implementer perspectives

Study implementers were prompted to reflect on the calls’ impact on study awareness and related implementation activities. However, no clear conceptual boundaries between these two concepts emerged in analysis of their responses. Thus, “implementation activities” was used to encompass study engagement/awareness and implementation activities. Implementers’ assessment of the check-in calls’ impact fell into three categories: (1) the calls had no effect on any implementation activities, (2) the calls served as a reminder about study participation (with no distinguishable impact on clinic activities), and (3) the calls led to changes in clinic engagement and activities through increasing the implementers’ sense of accountability.

#### No changes

Two of the 19 implementers (11%) said the phone check-ins had no impact on any implementation activities. One thought this was because the existing implementation infrastructure at their clinic was strong enough that the check-in calls had no relative impact; the other, because their clinic did not prioritize participation in the parent study: “I can’t say that it really affected our practice, just because we, you know, we had other bigger fish to fry, unfortunately” (Study implementer 1).

#### Reminder about study participation

Seven implementers (37%) said the phone check-ins reminded them that they were taking part in a research study, but could not describe if or how the calls impacted specific resultant actions. Two said the check-ins served as reminders, with one noting: “You know, it keeps me aware. …But how that spreads to the rest of the clinic, I think it’s definitely hard because there’s also so many other things going on that, you know, I think with the calls it’s definitely a helpful reminder, probably” (Study implementer 2). Another said the calls helped her retain focus on the research project more than she might have without them; another, that the calls helped him reflect on the implementation-related activities he had engaged in. One said the calls helped him reflect on decision support tools in general. In these responses, impact of the calls was foremost described as cognitive, and not related to tangible activities.

#### Changes in implementation activities through increased accountability

Ten study implementers (53%) noted that the phone check-ins had spurred implementation activities, all of whom referred to an increased sense of accountability resulting from engaging in the calls. For example, one said the check-ins motivated her to proactively pursue implementation activities so that she could report progress: “Yes, I do think you calling actually helped me to push a little bit more. Because I knew I had to come back to you and have some type of comment prepared for you. And if I’m not doing the work then I will have to report it back. So if there was no monitoring... Not monitoring, but no checks, no checking in, if that hadn’t been present I think it would have been easier to just slip away from doing it the proper way” (Study implementer 3). Another respondent described preparing for the check-ins by reviewing data reports related to cardioprotective prescribing. Another described reviewing reports after the check-in calls: “I’d say like after half of them [phone calls], I’ll go check numbers and look at things or see…if something has dropped off. I think if there wasn’t calls it would be pretty easy for it to fall by the wayside. […] But I think like in a busy place, like definitely, for sure I know it helps me” (Study implementer 4).

Some study implementers described engaging with other staff members about study activities as a result of the phone check-ins. One said: “Hey, we’ve got this project due, you know, coming from an outside source. So that when I see it I’m like, oh shoot, we didn’t do anything with the…[chuckles]…CVD bundle this month. But I definitely think … especially in the beginning to … have had those calls to keep things fresh and keep discussions open with you guys, but also within the other people, other managers here at [---] as far as, you know, what other people are doing. So I definitely think they’ve been helpful” (Study implementer 5). Another interviewee said that the check-ins reminded them to monitor the progress of all staff and to connect with them other about possible questions about the project: “Well, at least for me I consider every check-in time that we have scheduled like, okay, now, you know. I’m going to follow-up with [name researcher], let me see what my report says. Let me see if they have any questions, just so I can provide to you. So it does kind of alert me to like, oh, don’t forget about the statins” (Study implementer 6).

### Researcher perspectives

Like the implementers, the researchers perceived that the phone check-ins both served as reminders and encouraged some implementers to further engage in implementation-related activities. Overall, the researchers perceived that this impact fell into several overlapping categories, which generally aligned with the implementers’ perceptions.

#### Reminder about study participation

The arm 1 and arm 2 clinics had little interaction with the study team over most of the intervention period, other than the phone check-ins. Thus, the research team perceived that the check-in calls may have served to remind some implementers that they were taking part in a study: “So I think the awareness thing was probably the biggest. They weren’t hearing very much from us. So if nothing else, I think it kept people, this idea like three years in, oh right, there is this thing called SpreadNet” (Researcher 1).

#### Implementation activities

##### Documentation of study activities

Interviewers perceived that the check-in calls encouraged improved documentation of study activities in some cases. In routinely asking the implementers about time spent on activities related to implementing the CVD Bundle since the last call, they noticed that some implementers started to anticipate this question and prepared ahead of the calls to describe the activities they engaged in and the time spent on them. This improved the quality of the data the researchers were able to collect.

##### Engagement in study activities

The researchers also noted that some study implementers described an uptick in study activities, commonly immediately preceding and following the phone check-ins, but also at other times: “I think because of the calls probably [clinic name] tried to do more at the very beginning. They actually had some meetings. And I actually joined a phone meeting with them just to listen in a little bit. And they thought they were gonna do more than they ended up doing. But I’m not sure if they would have even had that meeting if we hadn’t been calling them and sort of asking them how they were doing” (Researcher 2). Researchers felt that the increased engagement in implementation activities was due to a heightened sense of motivation and accountability fostered by the phone check-ins. They also perceived that having calls over 35 months forged personal connections between some study staff and study implementers, and these relationships encouraged study implementers to engage in an increased level of study activities.

##### Requests for support with study activities

At the check-in calls, the researchers also regularly received questions about study expectations, other sites’ performance, the CVD Bundle, and how to use the EHR: “You know, they would just say like either…either these tools aren’t working or I don’t understand what this is, or what are other people doing? Or, you know, is this what you want? You know, and so… well, what are we allowed to tell them, you know. [Chuckles] How much help can we give? … And so that was something we were especially struggling with at the beginning I think, how to do that and be respectful. How to hold the party line” (Researcher 1).

In accordance with the study design, the researchers conducting the check-ins could not provide help or advice themselves. They referred questions from arm 3 clinics to the practice coach providing practice facilitation for these sites. All others were referred to the resources they previously had received, and to OCHIN’s technical support staff. This sometimes felt difficult to the researchers: they were the only study staff many implementers were regularly in touch with, and recognizing that some implementers were dedicating considerable resources to study participation, redirecting their requests for help felt uncomfortable and awkward.

## Discussion

These results suggest that collecting data through a series of phone check-ins in the context of implementation science research may incur unintended consequences: here, the check-ins were perceived to have had some impact on the implementers’ awareness of the study and related implementation activities, but the implementers did not differentiate between these two impacts. The importance of collecting qualitative data to assess the effects of contextual factors on outcomes in implementation studies is widely recognized [34]. However, these findings suggest that researchers should consider the potential for qualitative data collection to have unintended effects on implementation activities, and possibly on study outcomes, and underscore the complexities of capturing such data in a minimally impactful manner.

Notably, these findings suggest that although the phone check-ins were designed for data collection, they may, in some instances, have inadvertently served as an implementation strategy. Many implementers said the regular check-in calls created a sense of accountability. Accountability is widely considered important in quality improvement activities [35, 36], though it is commonly operationalized on an organizational level—e.g., through policy mechanisms [37, 38]. For individuals, several elements usually have to be present to incur a sense of accountability: the possibility of future evaluation, potential consequences of such an evaluation, and an external audience for relevant/reported behaviors [39]. Here, study implementers were aware that study results would be evaluated, and the researchers served as an external audience that monitored both behaviors and outcomes. In a similar vein, some study implementers perceived the check-in calls as a monitoring activity and described that it increased their study engagement, even though the calls were designed to be a neutral data collection method. This resonates with prior research demonstrating that monitoring via strategies such as audit and feedback can yield small but positive improvements in practice [40, 41]. Research on measurement reactivity also indicates that prompting research participants to reflect on a behavior can result in behavior changes and introduce bias to clinical trial results [13, 42, 43].

Researchers perceived that the relationships they developed with study implementers both shaped the quality of data collected and impacted study implementers’ engagement in implementation activities. Prior research indicates that positive relationships between researchers and participants can facilitate study recruitment [44], data sharing [45], and knowledge-sharing practices [46]. That such relationships can impact data collection and implementation activities is previously unreported in implementation science, warranting further investigation.

Accounting for and documenting unintended consequences of data collection activities may be possible by practicing reflexivity, a process used in the social sciences involving reflecting on one’s values, opinions, and underlying assumptions, and how they shape the research process and interactions with participants [47, 48]. In nursing and social work research, reflexivity has been used to create transparency and improve research quality [49–51]. Reflexive discussions among implementation researchers may also help with understanding and accounting for the unintended consequences of their data collection methods [24]. In implementation science, it may be difficult to engage study participants in data collection without incurring such consequences; critical self-reflection among researchers may yield greater transparency. Similarly, encouraging study implementers to reflect on any effects of data collection processes on study outcomes may help account for such effects.

Overall, these results emphasize the need to better understand the impact of qualitative data collection methods in implementation research. Phone check-ins may introduce bias to implementation trials if possible effects are not accounted for. They also suggest that check-in calls, such as those used here for data collection, could also be a useful tool to support practice change. For example, as discussed here, interviewees’ questions about the CVD Bundle prompted some adjustment to the implementation support they received (e.g., they were referred to the practice coach); this may have had a positive impact on the main study results [30]. Regularly scheduled phone check-ins, diaries, online logs, or reports might help create a sense of accountability in settings where behavior change is desired. Thus, one approach to mitigating the potential impacts of data collection methods on study outcomes might be to embrace it: to design data collection calls to include reminders about goals and expectations, allow room for questions and answers, or provide skills trainings—that is, to explicitly build the data collection into the implementation activities.

### Limitations

This analysis was not originally part of the parent trial; relevant data collection began after researchers observed unplanned effects of the phone check-ins, so some potentially useful data may have been missed. Further, not all researchers who participated in the data collection calls were available for interviews. Finally, more research is needed to assess the effects of data collection methods in implementation science, and the optimal balance between the cost of certain methods and the quality of the data that they yield.

### Conclusion

These results illustrate the potential impact of qualitative data collection on implementation activities during implementation science trials. Mitigating any such effects may prove challenging, but acknowledging and/or embracing such consequences could enhance the implementation of healthcare interventions. This work is presented to stimulate debate about the complexities of capturing data on implementation processes using common qualitative data collection methods.

## Data Availability

The qualitative data analyzed during the current study are not publicly available due to them containing information that could compromise research participant privacy; the codebook and data collection tools are available on request.

## Abbreviations

CHC: Community health center
CVD: Cardiovascular disease
EHR: Electronic health record
